# Systemic Manifestations of the Periodontal Disease: A Bibliometric Review

**DOI:** 10.3390/molecules25194508

**Published:** 2020-10-01

**Authors:** Paras Ahmad, Anas Imran Arshad, Elena Della Bella, Zohaib Khurshid, Martin Stoddart

**Affiliations:** 1 AO Research Institute Davos, 7270 Davos Platz, Switzerland; paras.ahmad@aofoundation.org (P.A.); elena.dellabella@aofoundation.org (E.D.B.); 2 Oral Medicine Unit, School of Dental Sciences, Universiti Sains Malaysia, Kota Bharu 16150, Kelantan, Malaysia; 3 Paediatric Dentistry Unit, School of Dental Sciences, Universiti Sains Malaysia, Kota Bharu 16150, Kelantan, Malaysia; anas.i@live.com; 4 Paedodontics Department, Rashid Latif Dental College, Lahore 54600, Pakistan; 5 Department of Prosthodontics and Dental Implantology, College of Dentistry, King Faisal University, Al-Ahsa 31982, Saudi Arabia; drzohaibkhurshid@gmail.com

**Keywords:** citation analysis, bibliometric analysis, top cited, periodontitis, systemic complications

## Abstract

This bibliometric review aimed to identify and analyze the top 100 most-cited publications on the systemic manifestations of periodontal disease (PD). A literature search was performed using the Web of Science (WoS) ‘All Databases’, without any restriction of language, publication year, or study design. Of 4418 articles, the top 100 were included based on their citation count. After downloading the full texts, their bibliometric information was extracted and analyzed. The citation counts for the top 100 articles ranged from 156 to 4191 (median 217). The most productive years were 2003 and 2005, with 20 articles on the list. Majority of the articles were published in the *Journal of Periodontology* (n = 25). The top 100 articles were generated primarily from the USA (n = 61). Most of the publications were clinical trials (n = 27) and focused on the cardiovascular manifestations of PD (n = 31). Most of the articles were within the evidence level V (n = 41). A total of 58 studies received funding and the most frequently used keyword in the top articles was “periodontal disease” (n = 39). The current citation analysis presents insights into the current trends in the systemic manifestations of periodontal disease.

## 1. Introduction

Periodontal disease is a chronic inflammatory microbial disease that directly damages the tooth-supporting structure (gingiva, periodontal ligament, alveolar bone, and cementum of the tooth) [1]. The sequelae of the periodontal disease are to gum swelling, gum bleeding, proceed to recession of junctional epithelium to apical loss of clinical attachment, and finally, pocket formation [2]. All these manifestations take time to develop and can be reversible, depending on the preventive strategies and intervention of the treatment [3]. Periodontal diseases are brought about by the host inflammatory response towards the virulence of the local biofilm [4]. Several modifiable factors include smoking, poor oral hygiene, female hormonal changes, diabetes mellitus, stress, and medications, with non-modifiable factors such as age, and hereditary factors, all of which predispose the host to periodontal diseases. The association of periodontal disease with systemic conditions were explored by various observational studies and clinical trials [5,6,7,8]. An association of different systemic conditions was documented on several occasions, which include cardiovascular diseases [9], renal problems [10], pregnancy-related issues [11], joint-related diseases [12], respiratory diseases [13], oncological predisposition [14], stroke [15], and diabetes [16]. Kinane et al. [17] suggested that periodontal diseases contributed to an increase in the total inflammatory and infectious disease burden, which could, in turn, lead to cardiovascular diseases and stroke. An exploratory investigation of atherosclerotic plaque composition was performed to hypothesize a biological origin that revealed the presence of bacterial DNA in a few studies [18,19]. However, other studies also negated this theory, which led to an uncertainty of the exact phenomenon [20,21]. Poor maternal periodontal health is correlated with low birth weight and pre-term babies [22,23,24]. Chronic periodontitis might lead to systemic inflammation as a response to the increased C-reactive protein levels. Increased levels of C-reactive proteins are associated with pre-term babies [25]. A review of previous cross-sectional studies and systematic reviews suggested that dental plaque might provide harbor to bacteria associated with respiratory tract infections, which could then be aspirated into the lungs to cause pneumonia [26]. A bidirectional association between periodontal diseases and diabetes was reported. Poorly controlled blood sugar levels were associated with periodontal conditions [27,28]. Increased levels of pro-inflammatory cytokines were noted in patients suffering from chronic periodontitis, which might then lead to insulin resistance, resulting in poor glycemic control [29,30,31]. *P. gingivalis* is one of the various periodontal pathogens, which is suspected of playing a role in the pathogenesis of rheumatoid arthritis [12,32,33]. The risk of developing systemic conditions is therefore increased with poor oral health, and a comprehensive healthcare system ensures healthy longevity.

*“If I have seen further, it is by standing on the shoulders of giants.”*

*-Isaac Newton, Letter to Robert Hooke, 1675*

This inspirational saying by Sir Isaac Newton [34] reflects the importance of past studies in science. Earlier studies are acknowledged by citing them in the modern research literature. Prior research and citation play a vital role in the evolution of knowledge [35]. Citation analysis is a bibliometric method to identify articles with the greatest impact on research and the clinical community, in a given discipline [36], providing the foundation for developing new research lines, techniques, and theories. This method was adopted in several areas of medicine such as urology [37], neuroscience [38], orthopedic trauma and surgery [39], suicidology [40], pediatrics [41], epilepsy [42], critical care medicine [43], and Parkinson‘s disease [44]. It is also used in different subfields in like dentistry, oral surgery, and medicine, including endodontics, orthodontics, periodontology, implant dentistry, prosthodontics, oral and maxillofacial surgery, dental traumatology, caries, squamous cell carcinoma, oral submucous fibrosis, oral leukoplakia, cleft lip and palate, and medication-related osteonecrosis of jaw (MRONJ) [45,46,47,48,49,50,51,52,53,54,55,56,57,58]. The definition of a “classic article” is a controversial topic across disciplines. The most suggested criterion is the securing of a certain citation count, for instance, at least 400 citations [36,38,44]. However, a publication that accomplished 100 or more citations could also be termed as a “classic publication,” depending upon the field under consideration, such as dentistry [59].

In periodontics, several bibliometric analyses were conducted [48,60,61]; however, no citation analysis of the most-cited “classic” articles published on the systemic manifestations of periodontal disease was performed. This study aimed to identify the top 100 most-cited “classic” articles published on the systemic complications of periodontal disease and to conduct an updated analysis to identify their fluctuating trends.

## 2. Results

### 2.1. Citation Count, Citation Density, and Current Citation Index

The primary characteristics of the top 100 most-cited articles are shown in Supplementary Table S1. The citation count of the top 100 publications varied from 156 to 4191 (median, 217), with a total citation count of 34,086. The most cited article, with a total of 4191 citations, was titled “Periodontal Disease in Pregnancy II. Correlation between Oral Hygiene and Periodontal Condition” [62], and was published in the Acta Odontologica Scandinavica. Its citation density (average citation count received per annum) was 76.20, with the current citation index (CCI) (number of citations received in 2019) of 177. The second most cited article, with a total of 4053 citations, was similar to the first article but was published one year earlier, titled “Periodontal disease in pregnancy I. Prevalence and severity” [11], and was published in the Acta Odontologica Scandinavica. Its citation density was 72.38, with the CCI of 140. The third most cited article, with a total of 903 citations, was titled “Periodontal disease and cardiovascular disease” [63] and was published in the Journal of Periodontology. Its citation density was 39.26, with the CCI of 15. According to the CCI 2019, the top-ranked article was the clinical trial published in 1964, securing 177 citations [62]. The second-ranked article was a literature review written by Hajishengallis G in 2015, with 169 citations [64]. The third-ranked article was the 1963 review, which counted 140 new citations [11]. As per citation density, the review by Hajishengallis had the highest score [64], with the second- and third-ranked articles being the clinical trials by Silness and Löe, with a citation density of 76.20 and 72.38, for [62] and [11], respectively.

**Figure 1:**
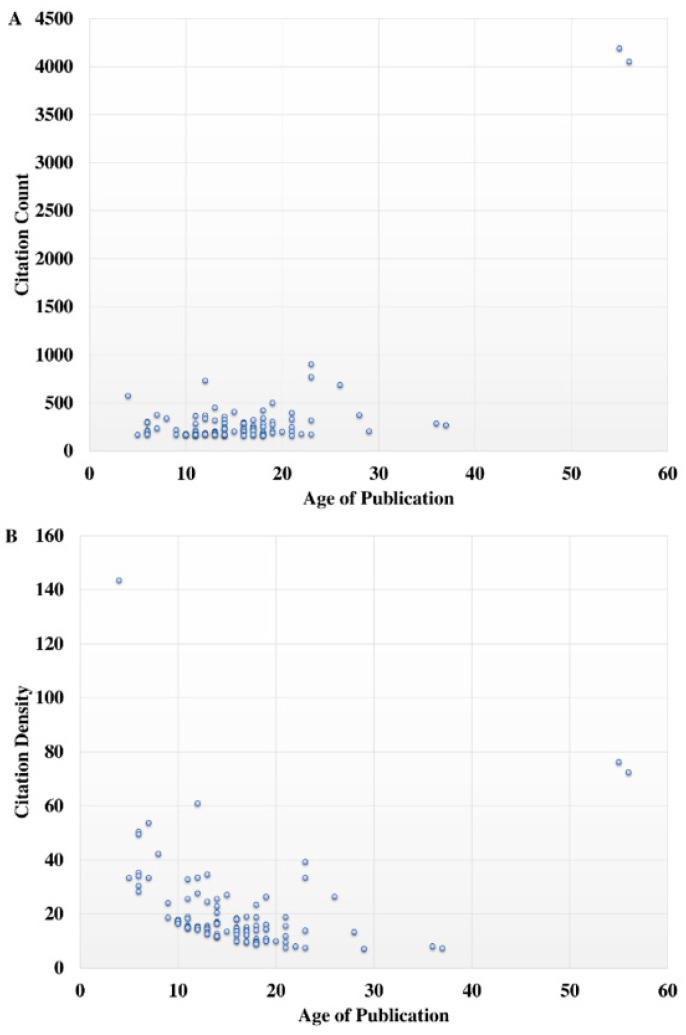
Association of (**A**) citation count and (**B**) citation density with age of publication.

According to the Shapiro–Wilk test, the distribution of data regarding citation count, citation density, and article age were not normal (*p* < 0.001). A non-significant trend towards a higher citation count with article age was observed (r = 0.009, *p* = 0.868) (Figure 1). However, a significant negative trend towards an increased citation density with the age of publication was observed (r = −0.440, *p* < 0.001) (Figure 1).

### 2.2. Distribution by Year

**Figure 2:**
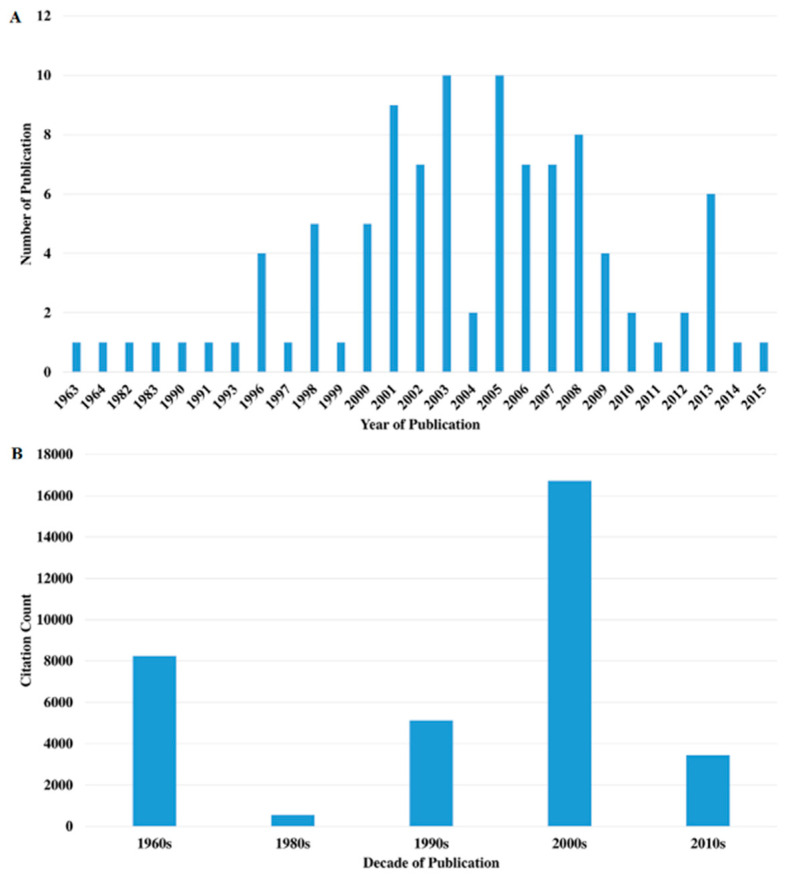
Citation analysis of the top 100 most-cited articles over the (**A**) years and (**B**) decades.

The top 100 most-cited articles were published between 1963 [11] and 2015 [64] (Figure 2). The most prolific years in terms of publications were 2003 and 2005, with ten publications each, followed by 2001 (n = 9). The year with most citations was 1964, with 4191 citations, followed by 1963 and 2003, with 4053 and 2308 citations, respectively. The decade with most publications (n = 69) and citations (n = 16,723) was the 2000s (Figure 2).

### 2.3. Contribution of Authors

**Figure 3:**
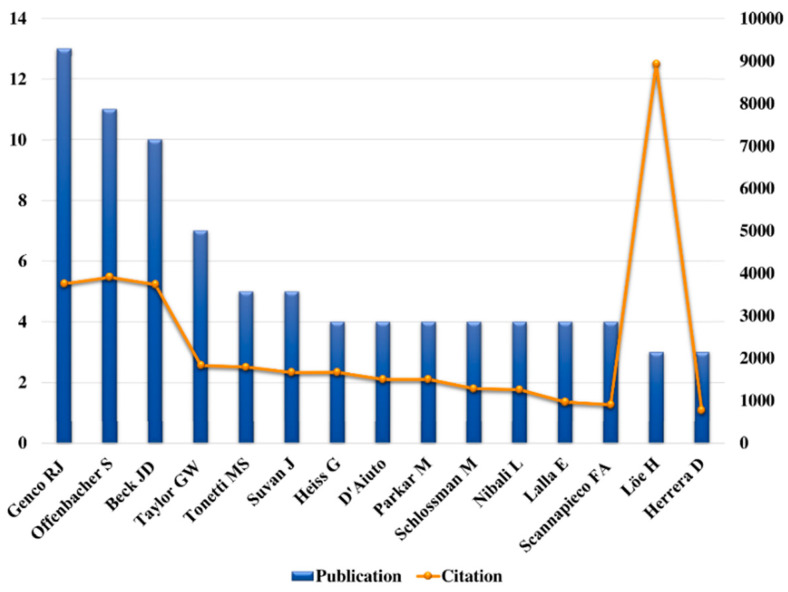
Citation analysis of the authors who contributed to the top 100 most-cited articles.

Many articles (n = 76) had between one and six authors, but publications with more than six authors were the most common (n = 24). The majority of the contributions were made by Genco (n = 13, 3750 citations), followed by Beck (n = 10, 3735), Offenbacher (n = 11, 3910), Taylor (n = 7, 1831), Tonetti (n = 5, 1793), and Suvan (n = 5, 1664) (Figure 3).

### 2.4. Contribution of Countries and Institutions

The top 100 most-cited publications originated from 19 countries, including Austria, Australia, Brazil, Canada, Chile, Denmark, Finland, Germany, Italy, Israel, Japan, Netherlands, New Zealand, Norway, Spain, Sweden, Turkey, the United Kingdom, and the United States of America (Table 1). According to the number of publications, most of the articles originated from the United States of America (n = 61, 17,037 citations), followed by the United Kingdom (n = 9, 2745), Netherlands (n = 3, 1003), Chile (n = 3, 723), Australia (n = 3, 615), Germany (n = 3, 574), Finland (n = 3, 515), Spain (n = 2, 392), Sweden (n = 2, 355), Brazil (n = 2, 332), Norway (n = 1, 4191), Denmark (n = 1, 4053), New Zealand (n = 1, 332), Italy (n = 1, 297), Turkey (n = 1, 229), Canada (n = 1, 198), Japan (n = 1, 173), and Israel (n = 1, 157).

There was a total of 55 institutions with which the corresponding authors were affiliated. The most prolific institution, with 13 publications, was Department of Oral Biology, School of Dental Medicine, University at Buffalo, USA, followed by Department of Periodontology, School of Dentistry, University of North Carolina at Chapel Hill, USA (n = 9) and Division of Periodontics, College of Dental Medicine, Columbia University, USA (n = 6) (Table 1).

### 2.5. Journal of Publication

The top 100 most-cited articles were published in both specialized and comprehensive periodicals (n = 36) (Table 2). The journal with the most number of publications was the Journal of Periodontology (n = 23), followed by the Annals of Periodontology (n = 10), the Journal of Clinical Periodontology (n = 9) and the Journal of Dental Research (n = 6). Acta Odontologica Scandinavica had the highest citation count (n = 8409), followed by the Journal of Periodontology (n = 8011), Annals of Periodontology (n = 2753), Journal of Clinical Periodontology (n = 1597), and Journal of Dental Research (n = 1592). The impact factors of journals ranged from 0.920 (Oral Health and Preventive Dentistry) to 74.699 (New England Journal of Medicine).

According to the Spearman-rank test, a statistically significant trend (*p* < 0.024) was observed between a journal age and the number of “classic” articles published in that journal. However, a statistically non-significant trend (*p* = 0.204) was observed between the impact factor of the journal and the number of “classics” published in that journal.

### 2.6. Topic of Publication

According to the topic of the article, the majority of the topic covered by the top 100 publications were the association of periodontal disease with cardiovascular diseases (n = 31) (8598 citations) and association of periodontitis with diabetes mellitus (n = 29) (7660 citations), followed by the systemic complications of periodontitis (mixed manifestations) (n = 14) (3918 citations), pregnancy-related manifestations (n = 11) (10,909 citations), rheumatic (n = 10) (1955 citations), pulmonary (n = 2) (399 citations), cerebrovascular diseases (n = 2) (478 citations), and cancer (n = 1) (169 citations) (Table 3). No statistical significance was detected (*p* = 0.724) while analyzing the median difference in the citation count per publication, between periodontitis and cardiovascular diseases—221.5 (156–903), periodontitis and diabetes mellitus—215 (156–686), periodontitis and systemic complications—234.5 (163–574), periodontitis and pregnancy-related manifestations—319 (157–4191), periodontitis and rheumatic diseases 187 (164–251), periodontitis and pulmonary diseases—199.5 (180–219), periodontitis and cerebrovascular diseases—239 (174–304), and periodontitis and cancer—169 (169).

### 2.7. Methodological Design of Publication

The most common methodological design in the top 100 publications was clinical trial (n = 27) (15,792 citations), followed by literature review (n = 26) (6609 citations), randomized controlled trial (n = 12) (3567 citations), survey (n = 10) (2076 citations), case-control study (n = 8) (1695 citations), systematic review (n = 6) (1505 citations), meta-analysis (n = 3) (829 citations), systematic review and meta-analysis (n = 3) (792 citations), animal study (n = 3) (630 citations), and consensus report (n = 2) (600 citations) (Table 3). No statistical significance was detected (*p* = 0.608) while analyzing the median difference in the citation count per publication among clinical trials—242.4 (156–4191), literature review—193.5 (157–574), randomized controlled trials—198 (163–731), survey—200 (156–258), case-control study—207 (165–327), systematic review—234.5 (204–319), meta-analysis—295 (176–358), systematic review and meta-analysis—272 (182–338), animal study—172 (171–287), and consensus report—300 (297–300).

### 2.8. Evidence Level of Publication

The top 100 most-cited publications could be classified into all evidence levels (ELs). Most of the articles were within evidence level V (n = 35), followed by EL III (n = 29), EL I (n = 15), EL II (n = 14), and EL IV (n = 7). Among these ELs, the total citation counts (r = −0.208, *p* = 0.082) and the citation density (r = 0.080, *p* = 0.336) did not vary significantly.

### 2.9. Keywords

**Figure 4:**
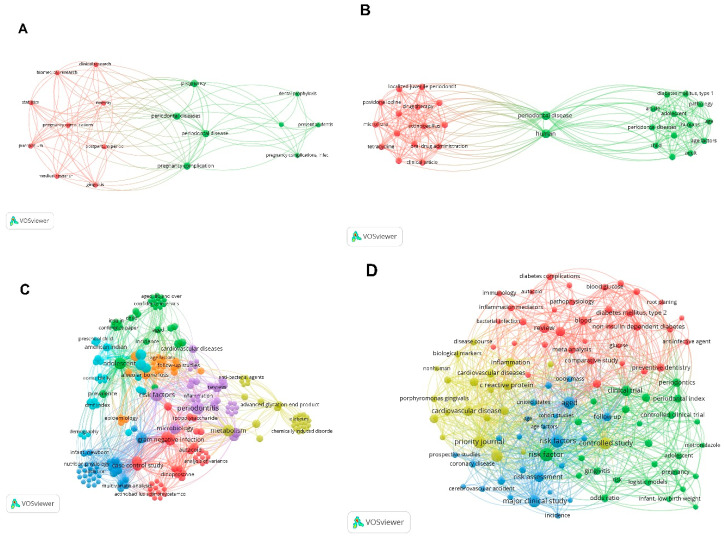

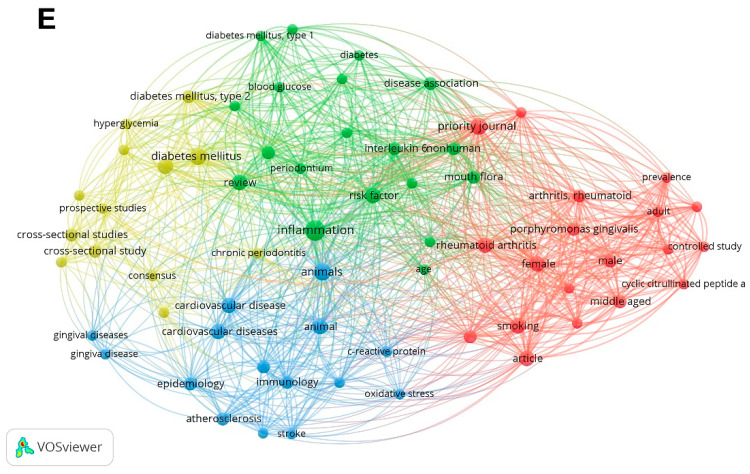
The distribution of the keywords over (**A**) 1960s, (**B**) 1980s, (**C**) 1990s, (**D**) 2000s, and (**E**) 2010s. The size of the node represents the frequency of the keyword used.

Out of the top 100 most-cited publications, only 76 articles contained keywords. A total of 182 keywords were identified. The most commonly used keyword was periodontal disease (n = 39), followed by periodontitis (n = 38), risk factors (n = 18), inflammation (n = 15), epidemiology (n = 14), diabetes mellitus (n = 14), atherosclerosis (n = 12), etiology (n = 11), and infection (n = 10). The distribution of the keywords over the decades is shown in Figure 4.

## 3. Discussion

This study aimed to identify the top 100 most-cited publications associated with the systemic manifestations of periodontal diseases and to analyze their primary bibliometric characteristics. Bibliometric analysis allows readers to gain historical insight and development of a particular specialty, by identifying and analyzing the most-cited publications that could assist researchers in understanding the emerging themes and future trends for a particular discipline [65,66]. For instance, the number of citations a publication receives could indicate other researchers’ interest in using the information for their own research. Highly cited articles could display a tendency in clinical practice and might, therefore, be considered to produce greater research and clinical interest in the reported disciplines [60]. Being the “most-cited” article, reflects its more frequent contribution to the studies published afterward; however, this characteristic alone does not provide sufficient information regarding its current impact and scientific quality as the main motive of citers if the selection of reference establishes the utility within research rather than scientific quality [67,68,69]. According to the definitions of “classic article” [70], a total of 354 (8%) articles were published on the systemic manifestations of periodontal diseases that achieved over 100 citations. Hence, all articles included in this study could be regarded as “classic articles.”

As our results depicted a fluctuating trend that was recently observed with regards to the contribution of international authors. For instance, during the 1990s, 86% of authors belonged to the institutions hailing from the US. This trend decreased significantly from 86% to 53% during the 2000s and again escalated recently during the 2010s from 53% to 71%. After the US, European countries, including the Netherlands and the UK, were prominent in this list of contributing authors. In addition to this study, several other bibliometric analyses reported that authors from Asia, Africa, and the Middle East, whether they were the first or the corresponding author, made a negligible contribution that could be considered a “classic” article [53,71,72,73,74]. Potential reasons might include language barriers, gaps in conducting research, and professional networking, as well as limited information access [75]. International organizations such as the World Health Organization and the United Nations could play a vital role in bolstering these healthcare developments.

As with several “most-cited” publications in medical and dental fields, this study reported that most of the most-cited articles originated from the United States. This significant contribution could be attributed to a larger scientific population, active researchers, and ample financial resources [46,53,72,74,76,77,78,79]. In addition to an unparalleled research work, an increased tendency among authors to cite articles originating from the US was observed [53,80]. It is noteworthy that first and second-ranked articles in the present study originated from the institutions hailing from Scandinavia, i.e., Norway and Denmark, respectively. As per the total citation count received by the top 100 articles, Norway and Denmark secured the second and third rank, despite their small population size. Importantly, a lack of multicenter studies was noticeable, as only the 13 most-cited articles had international collaborations, reflecting a need to escalate international collaboration.

Not surprisingly, the top 100 most-cited papers were authored in an array of specialties and were published in 35 different journals, indicating the multidisciplinary nature of research on systemic complications of periodontal diseases. Interestingly, approximately one-third of the top 100 highly cited articles were published in journals that are not necessarily dedicated to periodontitis and its systemic manifestations, including Circulation, Nature Reviews Immunology, and Journal of Dental Research. This might reflect a trend among authors to publish their research in high-impact journals to direct them to a broader audience.

In many bibliometric studies, it was reported that relevant studies were distributed among journals in accordance with Bradford’s law [81,82,83]. According to this bibliometric law, a few prolific journals account for a considerable percentage of all publications in a given discipline [84]. The studies published in these core journals are more likely to be referred to most commonly by successive articles [85]. Hence, the Journal of Periodontology, Annals of Periodontology, and Journal of Clinical Periodontology could be considered to be the core journals in this discipline, as approximately half of the most-cited articles were published in these (n = 45). Interestingly, in this study, the journal distribution pattern of the most-cited publications did not completely fit this law, as the list features almost negligible number of top-cited articles published by Periodontology 2000 and Journal of Periodontal Research, which are said to be one of the most prolific journals in the field of periodontics. Hence, the application of this law for conducting bibliometric analysis in some disciplines might cause inaccurate inferences.

In this study, no statistically significant association was found between the number of the most-cited articles published in a journal and the impact factor of that journal. This finding was in accordance with the findings of some bibliometric studies [86,87], but was contrary to those of several others [68]. This lack of association reflects that high-quality research might be highly cited, even if it is published in a relatively low-impact-factor journal [88]. In the current study, most articles (n = 52) were published by four prestigious periodontics journals, highlighting the expected significance of specialty journals. However, a substantial amount of non-periodontics journals with a higher impact factor than the periodontics journals published a smaller number of the most-cited articles.

Most of the highly cited publications were concerned with cardiovascular manifestations of periodontal diseases and diabetes mellitus, with 60 of the most-cited articles covering these two topics. The reason articles about systemic complications of periodontitis such as pulmonary diseases, cerebrovascular disorders, and cancer were not common among the list of 100 highly cited publications might be related to several factors; (a) majority of included papers originated from the US and European countries where complications of periodontitis related to diabetes mellitus and cardiovascular disease (CVD) are more common and relevant [89]; (b) the characteristics of both CVD and periodontal disease are very similar; they are usually chronic, multifactorial, and common [90], (c) in the USA and worldwide, CVD accounts for around 50% and 29% of deaths per year and is considered to be the second leading cause of death preceded by parasitic and infectious diseases [91], (d) globally, more than 18 million population suffer from diabetes mellitus and its severe complications, including blindness, leg amputation, CVD, impaired wound healing, renal dialysis, and death [92]. These findings do not necessarily reflect that high-quality research about other systemic manifestations of periodontal diseases was not commonly performed; rather it might mean that such studies were not cited as many times as studies on other complications.

From 1990 to 1999, the hottest research topic was the association between periodontitis and diabetes mellitus. From 2000 to 2009, the trend shifted to cardiovascular-related manifestations of periodontal diseases. However, from 2010 to 2015, the relationship between periodontitis and diabetes mellitus re-emerged as the hottest research topic. This could be explained by the fact that the relationship between periodontal diseases and diabetes mellitus is a long-discussed research topic with conflicting conclusions. In the general population, both these diseases have a relatively high incidence (periodontitis 14% and diabetes 1% to 6%), as well as several common pathways in their pathogenesis [93].

Based on the hierarchy of research evidence level, systematic reviews, meta-analyses, and randomized controlled trials (RCTs) provide the highest quality of evidence [36]. A distinctive characteristic of this analysis was that it included 29 highest quality evidence level studies, including systematic reviews, meta-analyses, both systematic reviews, and meta-analysis, as well as RCTs. These findings did not coincide with the findings of several other bibliometric analyses performed on various specialties within dentistry and medicine [39,53,59]. Recently, these high evidence level studies were performed and secured high citations, despite only being published in recent years [94]. Such reports are useful for facilitating decision-making, directing practice, and advancing research, so a high amount of such studies as reflected in the current study is not surprising and provides further proof of maturation of the discipline [95].

This bibliometric analysis had several limitations. First, for a given research field, many factors might influence the citation count, including the age of the publication, journal of publication, reputation of author, institution, and country of origin as well as the original language. Second, the analysis of self-citations and citations in textbooks and lectures was not performed. Moreover, some authors might be inclined to cite articles from a particular journal in which they intend to publish an article [96]. Third, the analysis of the contributing countries and institutions was based on the address of the corresponding author. A statistical bias might occur once the address of the corresponding author is changed [97]. Furthermore, for corresponding authors working in multiple institutions, we only considered the first institution.

## 4. Conclusions and Future Recommendations

Citation analysis aids in providing useful and interesting information for several disciplines, including determination of current trends and assessment of future direction the research might take. This study revealed that the most common topic observed in the top-cited publications was the association of periodontal disease with cardiovascular diseases and diabetes mellitus. Interestingly, articles related to the systemic manifestations of periodontal diseases, including pulmonary and cerebrovascular diseases as well as cancer, were uncommon among the most-cited publications, which reflects that more research is required in these domains. Surprisingly, unlike several other bibliometric studies performed within dentistry and medicine, the number of clinical trials was higher than the review articles. However, systematic reviews and meta-analyses with evidence might be needed in the literature.

Overall, further understanding of the associations between periodontal disease and its association with systemic complications could raise society’s awareness of the links between inflammatory diseases and oral health. This could lead to large-scale and far reaching general medical benefits through periodontal intervention, including improving glycemic control in diabetics [98], potentially decreasing mortality and morbidity in individuals predisposed to respiratory diseases [99,100], improving endothelial function in CVD patients [101], and possibly alleviating the risk of alveolar bone loss in patients with osteoporosis [102,103,104].

## 5. Materials and Methods

### 5.1. Data Sources and Search Methodology

This study, being a bibliometric review, was exempt from the institutional ethics committee review. Preferred Reporting Items for Systematic Reviews and Meta-Analysis guidelines were used for data retrieval and reporting [94]. Articles on the systemic complications of periodontitis were gathered from the Clarivate Analytics’ Web of Science (WoS) (https://www.webofknowledge.com), considering the ‘All Databases’ section, which included Derwent Innovations Index, Russian Science Citation Index, Web of Science Core Collection, SciELO Citation Index, and KCI—Korean Journal Database. There was no restriction of publication year and language. Since the database is still functioning, to avoid daily updating bias, an extensive search was conducted on a single day, 2nd February 2020. The search was performed using the terms: “Periodontitis OR Periodontal disease AND Respiratory”, “Periodontitis OR Periodontal disease AND Pulmonary”, “Periodontitis OR Periodontal disease AND Pneumonia”, “Periodontitis OR Periodontal disease AND Cardiovascular”, “Periodontitis OR Periodontal disease AND Hypertension”, “Periodontitis OR Periodontal disease AND Coronary Heart”, “Periodontitis OR Periodontal disease AND Myocardial Infarction”, “Periodontitis OR Periodontal disease AND Angina Pectoris”, “Periodontitis OR Periodontal disease AND Stroke”, “Periodontitis OR Periodontal disease AND Atherosclerosis”, “Periodontitis OR Periodontal disease AND Endothelial”, “Periodontitis OR Periodontal disease AND Ischemic Heart”, “Periodontitis OR Periodontal disease AND Diabetes Mellitus”, “Periodontitis OR Periodontal disease AND Pregnancy”, “Periodontitis OR Periodontal disease AND Low Birth Weight”, “Periodontitis OR Periodontal disease AND Rheumatic Disease”, “Periodontitis OR Periodontal disease AND Osteoporosis”, “Periodontitis OR Periodontal disease AND Arthritis”, “Periodontitis OR Periodontal disease AND Kidney”, “Periodontitis OR Periodontal disease AND Renal”, “Periodontitis OR Periodontal disease AND Cerebrovascular”, “Periodontitis OR Periodontal disease AND Alzheimer’s”, “Periodontitis OR Periodontal disease AND Dementia”, “Periodontitis OR Periodontal disease AND Cancer”, “Periodontitis OR Periodontal disease AND Carcinoma”, “Periodontitis OR Periodontal disease AND Systemic”, and “Periodontitis OR Periodontal disease AND Focal Infection” in the title field.

### 5.2. Publication Selection

According to the selected database, 4418 articles were retrieved. After finalizing the list of the top 100 most-cited publications, the retrieved articles were arranged in descending order, based on their citation count. Then, the full text of included articles was downloaded and analyzed to extract the data of interest.

### 5.3. Data Extraction

The following bibliometric variables were retrieved from each publication identified—article’s title and age (i.e., 2019 minus the year of publication), citation count, citation density (average citation count received by an article per year) [105], current citation index (number of citation received in 2019) [59], authorship (name, contribution and amount), institution and country of origin, publication year, journal’s title, age (i.e., the year in which the first issue was published), impact factor (i.e., Journal Citation Report 2019), keywords, methodological design [106], the topic of the publication, and evidence level. In the case of publications with equal citation count, the ranking was done based on citation density and publications with higher citation density was ranked higher. Additionally, the institution and country of article’s origin was determined based on the address provided for the corresponding author.

The methodological design (literature review, systematic review, meta-analysis, systematic review and meta-analysis, clinical trial, randomized clinical trial, case-control study, animal study, consensus report, and survey) was based on the Cochrane Collaboration Glossary [106]. The thematic fields were categorized into respiratory diseases (chronic obstructive pulmonary disease and pneumonia), cardiovascular diseases (hypertension, coronary heart disease, myocardial infarction, angina pectoris, stroke, atherosclerosis, and ischemic heart disease), diabetes mellitus, pregnancy-related complications (low birth weight and pre-term low birth weight), rheumatic disorders (arthritis and osteoporosis), cerebrovascular diseases (Alzheimer‘s disease and dementia), cancer, and other systemic manifestations associated with periodontal diseases, including focal infection.

The Visualization of Similarities viewer software [107] was used to create collaboration network maps regarding the co-occurrences of all keywords.

### 5.4. Statistical Analysis

Descriptive and bivariate analyses were performed using a statistical software package, i.e., IBM SPSS Statistics version 24.0 (IBM, Chicago, IL, USA). To assess the normality of the data (citation count, citation density, and age of article), the Shapiro-Wilk test was conducted. Mean (standard deviation) or median (interquartile range) (topic and study design of article) were calculated based on normality and distribution of data. To evaluate the median differences between the independent groups, the Kruskal–Wallis test was performed (topic and study design of article). Any decrease or increase in the time-dependent trends was analyzed by performing the Mann–Kendall trend test. The Spearman-rank test was performed to assess the correlation between the publication count of the journal and the age of the journal. A value of *p* < 0.05 was considered statistically significant.

## Figures and Tables

**Table 1:** List of contributing countries and institutions to the most-cited publications.

Name of Country	No. of Publication
United States of America	61
United Kingdom	9
Netherlands	3
Chile	3
Australia	3
Germany	3
Finland	3
Sweden	2
Spain	2
Brazil	2
Norway	1
Denmark	1
New Zealand	1
Italy	1
Turkey	1
Canada	1
Japan	1
Austria	1
Israel	1
**Name of Institution**	
School of Dental Medicine, University at Buffalo, USA	13
School of Dentistry, University of North Carolina at Chapel Hill, USA	9
College of Dental Medicine, Columbia University, USA	6
School of Dentistry, University of Michigan, USA	4
National Institute of Dental Research, National Institutes of Health, USA	3
Academic Center for Dentistry Amsterdam (ACTA), The Netherlands	3
Eastman Dental Institute and Hospital, University College London, UK	3
Faculty of Dentistry, University of Chile, Chile	3

**Table 2:** List of journals that published the top 100 “classic” articles.

Journals (Abbreviation)	Impact Factor *	Journal Age (Years)	Number of Articles	Total Citation	Median Citation
J. Periodontol.	3.742	88	25	8011	251
Ann. Periodontol.	-	23	11	2753	240
J. Clin. Periodontol.	5.241	45	8	1597	178.5
J. Dent. Res.	4.914	100	7	1592	205
Arterioscler. Thromb. Vasc. Biol.	6.604	38	4	974	258.5
Acta Odontol. Scand.	1.573	80	3	8409	4053
Diabetes Care	16.019	41	3	1082	233
Am. Heart J.	4.153	94	3	728	195
Arch. Intern. Med.	17.333 **	111	3	664	197
J. Am. Dent. Assoc.	2.803	105	3	635	204
Oral Dis.	2.613	24	2	462	231
Circulation	23.603	69	2	461	230.5
Stroke	7.190	49	2	419	209.5
Arthritis Rheumatol.	9.586	61	2	401	200.5
Alzheimers. Dement.	17.127	14	1	174	174
Am. J. Epidemiol.	4.526	80	1	206	206
BJOG	4.663	117	1	319	319
Clin. Microbiol. Infect.	7.117	23	1	332	332
Community Dent. Oral Epidemiol.	2.135	46	1	258	258
Diabetologia	7.518	54	1	376	376
Eur. Heart J.	22.673	39	1	168	168
Int. Immunopharmacol.	3.943	18	1	164	164
JAMA	45.540	136	1	272	272
J. Am. Coll. Cardiol.	20.589	36	1	194	194
J. Cardiovasc. Risk	2.548 ***	-	1	198	198
J. Gen. Intern. Med.	4.597	33	1	365	365
J. Periodont. Res.	2.926	53	1	172	172
J. Rheumatol.	3.350	45	1	200	200
Lancet. Oncol.	33.752	19	1	169	169
Nat. Rev. Endocrinol.	28.800	14	1	338	338
Nat. Rev. Immunol.	40.358	18	1	574	574
Odontology	1.840	107	1	203	203
Oral Health Prev. Dent.	0.920	16	1	176	176
Oral Surg. Oral Med. Oral Pathol. Oral Radiol. Endod.	1.457 ^#^	58	1	295	295
Periodontol. 2000	7.718	26	1	186	186
N. Eng. J. Med.	74.699	107	1	731	731

* Journal Citation Report, 2019; ** Journal Citation Report, 2014, *** Journal Citation Report, 2005; ^#^ Journal Citation Report, 2011.

**Table 3:** Distribution of topics and methodological designs of the 100 most-cited articles.

Topic	No. of Article	Citation Count	Average Citation Count	Median Citation
Cardiovascular diseases	31	8598	277.35	217
Diabetes mellitus	29	7660	264.13	229
Collective systemic manifestations	14	3918	279.85	234.5
Pregnancy-related diseases	11	10909	991.72	319
Rheumatic diseases	10	1955	195.5	187
Cerebrovascular diseases	2	478	239	239
Respiratory diseases	2	399	199.5	199.5
Cancer	1	169	169	169
**Study design**				
Clinical trial	28	15788	574.85	249
Literature review	26	6606	254.08	194
Randomized controlled trial	11	3566	247.84	179
Survey	11	2076	216.12	204.50
Case-control study	7	1695	217.87	204
Systematic review	6	1504	253.83	237
Meta-analysis	3	829	279.66	297
Systematic review & meta-analysis	3	792	297.75	320
Animal study	3	630	210.66	173
Consensus report	2	600	303.50	303.50

## Supplementary Materials

The following are available online. Table S1: Ranking list of the top 100 most cited articles.
