# Supplementary material for: Systemic Manifestations of the Periodontal Disease: A Bibliometric Review
Source: Molecules. 2020 Oct 1;25(19):4508. doi: 10.3390/molecules25194508 (PMC7582719; doi:10.3390/molecules25194508)
Supplement: Supplementary file 1 [file molecules-25-04508-s001.pdf]

Table S1. Ranking list of the top 100 most cited articles

Sr No.	Title of article	Citation count (WOS)	Citation density	Current citation index (2019)	Evidence level
1	Silness J, Löe H. Periodontal disease in pregnancy II. Correlation between oral hygiene and periodontal condition. *Acta Odontol Scand*. 1964;22:121-135.	4191	76.20	177	III
2	Löe H, Silness J. Periodontal disease in pregnancy I. Prevalence and severity. *Acta Odontol Scand*. 1963;21:533-551.	4053	72.38	140	III
3	Beck JD, Garcia R, Heiss G, Vokonas PS, Offenbacher S. Periodontal disease and cardiovascular disease. *J Periodontol*. 1996;67:1123-1137.	903	39.26	15	III
4	Offenbacher S, Katz V, Fertik G, Collins J, Boyd D, Maynor G, McKaig R, Beck JD. Periodontal infection as a possible risk factor for preterm low birth weight. *J Periodontol*. 1996;67:1103-1113.	769	33.43	25	III
5	Tonetti MS, D'Aiuto F, Nibali L, Donald A, Storry C, Parkar M, Suvan J, Hingorani AD, Vallance P, Deanfield J. Treatment of periodontitis and endothelial function. *N Eng J Med*. 2007;356:911-920.	731	60.91	54	II
6	Löe H. Periodontal disease: the sixth complication of diabetes mellitus. *Diabetes Care*. 1993;16:329-334.	686	26.38	40	V
7	Hajishengallis G. Periodontitis: from microbial immune subversion to systemic inflammation. *Nat Rev Immunol*. 2015;15:30-44.	574	143.50	169	V
8	Loos BG, Craandijk J, Hoek FJ, Dillen PMWv, Van Der Velden U. Elevation of systemic markers related to cardiovascular diseases in the peripheral blood of periodontitis patients. *J Periodontol*. 2000;71:1528-1534.	500	26.32	21	III
9	Mealey BL, Oates TW. Diabetes mellitus and periodontal diseases. *J Periodontol*. 2006;77:1289-1303.	450	34.62	24	III
10	Noack B, Genco RJ, Trevisan M, Grossi S, Zambon JJ, De Nardin E. Periodontal infections contribute to elevated systemic C-reactive protein level. *J Periodontol*. 2001;72:1221-1227.	422	23.44	12	V
11	D’Aiuto F, Parkar M, Andreou G, Suvan J, Brett PM, Ready D, Tonetti MS. Periodontitis and systemic inflammation: control of the local infection is associated with a reduction	406	27.07	21	V

	in serum inflammatory markers. *J Dent Res*. 2004;83:156-160.				
12	Grossi SG, Genco RJ. Periodontal disease and diabetes mellitus: a two-way relationship. *Ann Periodontol*. 1998;3:51-61.	395	18.81	14	III
13	Preshaw P, Alba A, Herrera D, Jepsen S, Konstantinidis A, Makrilakis K, Taylor R. Periodontitis and diabetes: a two-way relationship. *Diabetologia*. 2012;55:21-31.	376	53.71	15	II
14	Emrich LJ, Shlossman M, Genco RJ. Periodontal disease in non-insulin-dependent diabetes mellitus. *J Periodontol*. 1991;62:123-131.	372	13.29	14	III
15	Bahekar AA, Singh S, Saha S, Molnar J, Arora R. The prevalence and incidence of coronary heart disease is significantly increased in periodontitis: a meta-analysis. *Am Heart J*. 2007;154:830-837.	368	33.45	22	I
16	Humphrey LL, Fu R, Buckley DI, Freeman M, Helfand M. Periodontal disease and coronary heart disease incidence: a systematic review and meta-analysis. *J Gen Intern Med*. 2008;23:2079.	365	32.82	29	I
17	Genco RJ, Grossi SG, Ho A, Nishimura F, Murayama Y. A proposed model linking inflammation to obesity, diabetes, and periodontal infections. *J Periodontol*. 2005;76:2075-2084.	358	25.57	10	I
18	Taylor GW. Bidirectional interrelationships between diabetes and periodontal diseases: an epidemiologic perspective. *Ann Periodontol*. 2001;6:99-112.	338	18.78	10	V
19	Lalla E, Papapanou PN. Diabetes mellitus and periodontitis: a tale of two common interrelated diseases. *Nat Rev Endocrinol*. 2011;7:738.	338	42.25	46	V
20	Seymour G, Ford P, Cullinan M, Leishman S, Yamazaki K. Relationship between periodontal infections and systemic disease. *Clin Microbiol Infect*. 2007;13:3-10.	332	27.67	16	IV
21	Offenbacher S, Jared H, O'reilly P, Wells S, Salvi G, Lawrence H, Socransky S, Beck JD. Potential pathogenic mechanisms of periodontitis-associated pregnancy complications. *Ann Periodontol*. 1998;3:233-250.	327	15.57	4	II
22	López NJ, Smith PC, Gutierrez J. Periodontal therapy may reduce the risk of preterm low birth weight in women with periodontal disease: a randomized controlled trial. *J Periodontol*. 2002;73:911-924.	322	18.94	6	V

23	Loos BG. Systemic markers of inflammation in periodontitis. *J Periodontol*. 2005;76:2106-2115.	320	22.86	29	I
24	Xiong X, Buekens P, Fraser W, Beck JD, Offenbacher S. Periodontal disease and adverse pregnancy outcomes: a systematic review. *BJOG*. 2006;113:135-143.	319	24.54	12	III
25	Taylor GW, Burt BA, Becker MP, Genco RJ, Shlossman M, Knowler WC, Pettitt DJ. Severe periodontitis and risk for poor glycemic control in patients with non-insulin-dependent diabetes mellitus. *J Periodontol*. 1996;67:1085-1093.	318	13.83	15	III
26	Wu T, Trevisan M, Genco RJ, Dorn JP, Falkner KL, Sempos CT. Periodontal disease and risk of cerebrovascular disease: the first national health and nutrition examination survey and its follow-up study. *Arch Intern Med*. 2000;160:2749-2755.	304	16.00	9	I
27	Chapple IL, Genco RJ. Diabetes and periodontal diseases: consensus report of the Joint EFP/AAP Workshop on Periodontitis and Systemic Diseases. *J Periodontol*. 2013;84:S106-S112.	303	50.50	47	I
28	Tonetti MS, Van Dyke TE. Periodontitis and atherosclerotic cardiovascular disease: consensus report of the Joint EFP/AAPWorkshop on Periodontitis and Systemic Diseases. *J Periodontol*. 2013;84:S24-S29.	297	49.50	51	I
29	Janket S-J, Baird AE, Chuang S-K, Jones JA. Meta-analysis of periodontal disease and risk of coronary heart disease and stroke. *Oral Surg Oral Med Oral Pathol Oral Radiol Endod*. 2003;95:559-569.	295	18.44	15	I
30	Scannapieco FA, Bush RB, Paju S. Associations between periodontal disease and risk for atherosclerosis, cardiovascular disease, and stroke. A systematic review. *Ann Periodontol*. 2003;8:38-53.	294	18.38	7	II
31	Beck JD, Eke P, Heiss G, Madianos P, Couper D, Lin D, Moss K, Elter J, Offenbacher S. Periodontal disease and coronary heart disease: a reappraisal of the exposure. *Circulation*. 2005;112:19-24.	287	20.50	10	V
32	Lalla E, Lamster IB, Hofmann MA, Bucciarelli L, Jerud AP, Tucker S, Lu Y, Papapanou PN, Schmidt AM. Oral infection with a periodontal pathogen accelerates early atherosclerosis in apolipoprotein E–null mice. *Arterioscler Thromb Vasc Biol*. 2003;23:1405-1411.	287	17.94	8	III

33	Slots J, Rosling BG. Suppression of the periodontopathic microflora in localized juvenile periodontitis by systemic tetracycline. *J Clin Periodontol*. 1983;10:465-486.	285	7.92	1	III
34	Beck JD, Elter JR, Heiss G, Couper D, Mauriello SM, Offenbacher S. Relationship of periodontal disease to carotid artery intima-media wall thickness: the atherosclerosis risk in communities (ARIC) study. *Arterioscler Thromb Vasc Biol*. 2001;21:1816-1822.	282	15.67	10	V
35	Taylor GW, Borgnakke W. Periodontal disease: associations with diabetes, glycemic control and complications. *Oral Dis*. 2008;14:191-203.	282	25.64	10	I
36	Hujoel PP, Drangsholt M, Spiekerman C, DeRouen TA. Periodontal disease and coronary heart disease risk. *JAMA*. 2000;284:1406-1410.	272	14.32	4	III
37	Cianciola L, Park B, Bruck E, Mosovich L, Genco R. Prevalence of periodontal disease in insulin-dependent diabetes mellitus (juvenile diabetes). *J Am Dent Assoc*. 1982;104:653-660.	268	7.24	3	V
38	Tsai C, Hayes C, Taylor GW. Glycemic control of type 2 diabetes and severe periodontal disease in the US adult population. *Community Dent Oral Epidemiol*. 2002;30:182-192.	258	15.18	12	III
39	Mercado F, Marshall RI, Klestov A, Bartold P. Relationship between rheumatoid arthritis and periodontitis. *J Periodontol*. 2001;72:779-787.	251	13.94	15	V
40	Beck JD, Offenbacher S, Williams R, Gibbs P, Garcia R. Periodontitis: a risk factor for coronary heart disease? *Ann Periodontol*. 1998;3:127-141.	247	11.76	2	V
41	Beck JD, Offenbacher S. Systemic effects of periodontitis: epidemiology of periodontal disease and cardiovascular disease. *J Periodontol*. 2005;76:2089-2100.	241	17.21	13	I
42	Haffajee AD, Socransky SS, Gunsolley JC. Systemic anti-infective periodontal therapy. A systematic review. *Ann Periodontol*. 2003;8:115-181.	240	15.00	14	III
43	López NJ, Smith PC, Gutierrez J. Higher risk of preterm birth and low birth weight in women with periodontal disease. *J Dent Res*. 2002;81:58-63.	238	14.00	8	III
44	Amar S, Gokce N, Morgan S, Loukideli M, Van Dyke TE, Vita JA. Periodontal disease is	235	14.69	11	III

	associated with brachial artery endothelial dysfunction and systemic inflammation. *Arterioscler Thromb Vasc Biol*. 2003;23:1245-1249.				
45	Scher JU, Ubeda C, Equinda M, Khanin R, Buischi Y, Viale A, Lipuma L, Attur M, Pillinger MH, Weissmann G. Periodontal disease and the oral microbiota in new-onset rheumatoid arthritis. *Arthritis Rheum*. 2012;64:3083-3094.	234	33.43	31	III
46	Saremi A, Nelson RG, Tulloch-Reid M, Hanson RL, Sievers ML, Taylor GW, Shlossman M, Bennett PH, Genco R, Knowler WC. Periodontal disease and mortality in type 2 diabetes. *Diabetes Care*. 2005;28:27-32.	233	16.64	15	I
47	Herrera D, Sanz M, Jepsen S, Needleman I, Roldán S. A systematic review on the effect of systemic antimicrobials as an adjunct to scaling and root planing in periodontitis patients. *J Clin Periodontol*. 2002;29:136-159.	229	13.47	10	II
48	Kıran M, Arpak N, Ünsal E, Erdoğan MF. The effect of improved periodontal health on metabolic control in type 2 diabetes mellitus. *J Clin Periodontol*. 2005;32:266-272.	229	16.36	9	I
49	Scannapieco FA, Bush RB, Paju S. Associations between periodontal disease and risk for nosocomial bacterial pneumonia and chronic obstructive pulmonary disease. A systematic review. *Ann Periodontol*. 2003;8:54-69.	219	13.69	12	III
50	Joshipura KJ, Hung H-C, Rimm EB, Willett WC, Ascherio A. Periodontal disease, tooth loss, and incidence of ischemic stroke. *Stroke*. 2003;34:47-52.	217	13.56	7	V
51	Kebschull A, Demmer R, Papapanou P. "Gum bug, leave my heart alone!"—epidemiologic and mechanistic evidence linking periodontal infections and atherosclerosis. *J Dent Res*. 2010;89:879-902.	217	24.11	20	V
52	Linden GJ, Lyons A, Scannapieco FA. Periodontal systemic associations: review of the evidence. *J Periodontol*. 2013;84:S8-S19.	212	35.33	31	IV
53	Pischon N, Pischon T, Kröger J, Gülmez E, Kleber BM, Bernimoulin JP, Landau H, Brinkmann PG, Schlattmann P, Zernicke J. Association among rheumatoid arthritis, oral hygiene, and periodontitis. *J Periodontol*. 2008;79:979-986.	209	19.00	26	V
54	Wu T, Trevisan M, Genco RJ, Falkner KL, Dorn JP, Sempos CT. Examination of the relation between periodontal health status	206	10.84	6	IV

	and cardiovascular risk factors: serum total and high density lipoprotein cholesterol, C-reactive protein, and plasma fibrinogen. *Am J Epidemiol*. 2000;151:273-282.				
55	Davenport E, Williams C, Sterne J, Murad S, Sivapathasundram V, Curtis M. Maternal periodontal disease and preterm low birthweight: case-control study. *J Dent Res*. 2002;81:313-318.	205	12.06	9	I
56	Borgnakke WS, Yl€ ostalo PV, Taylor GW, Genco RJ. Effect of periodontal disease on diabetes: systematic review of epidemiologic observational evidence. *J Periodontol*. 2013;84:S135-S152.	204	34.00	24	III
57	Shlossman M, Knowler WC, Pettitt DJ, Genco RJ. Type 2 diabetes mellitus and periodontal disease. *J Am Dent Assoc*. 1990;121:532-536.	204	7.03	9	V
58	Kim J, Amar S. Periodontal disease and systemic conditions: a bidirectional relationship. *Odontol*. 2006;94:10-21.	203	15.62	22	IV
59	Grau AJ, Becher H, Ziegler CM, Lichy C, Buggle F, Kaiser C, Lutz R, Bu¨ltmann S, Preusch M, Do¨rfer CE. Periodontal disease as a risk factor for ischemic stroke. *Stroke*. 2004;35:496-501.	202	13.47	8	V
60	Page RC. The pathobiology of periodontal diseases may affect systemic diseases: inversion of a paradigm. *Ann Periodontol*. 1998;3:108-120.	200	9.52	3	V
61	De Pablo P, Dietrich T, McAlindon TE. Association of periodontal disease and tooth loss with rheumatoid arthritis in the US population. *J Rheumatol*. 2008;35:70-76.	200	18.18	24	V
62	Morrison HI, Ellison LF, Taylor GW. Periodontal disease and risk of fatal coronary heart and cerebrovascular diseases. *J Cardiovasc Risk*. 1999;6:7-11.	198	9.90	0	V
63	Slade GD, Ghezzi EM, Heiss G, Beck JD, Riche E, Offenbacher S. Relationship between periodontal disease and C-reactive protein among adults in the Atherosclerosis Risk in Communities study. *Arch Intern Med*. 2003;163:1172-1179.	197	12.31	7	II
64	D'Aiuto F, Parkar M, Nibali L, Suvan J, Lessem J, Tonetti MS. Periodontal infections cause changes in traditional and novel cardiovascular risk factors: results from a randomized controlled clinical trial. *Am Heart J*. 2006;151:977-984.	195	15.00	4	II
65	Howell TH, Ridker PM, Ajani UA, Christen WG, Hennekens CH. Periodontal disease	194	10.78	6	V

	and risk of subsequent cardiovascular disease in US male physicians. *J Am Coll Cardiol*. 2001;37:445-450.				
66	Mercado F, Marshall RI, Klestov AC, Bartold PM. Is there a relationship between rheumatoid arthritis and periodontal disease? *J Clin Periodontol*. 2000;27:267-272.	187	9.84	8	V
67	Lerner U. Inflammation-induced bone remodeling in periodontal disease and the influence of post-menopausal osteoporosis. *J Dent Res*. 2006;85:596-607.	187	14.38	10	V
68	Mealey BL, Ocampo GL. Diabetes mellitus and periodontal disease. *Periodontol 2000*. 2007;44:127-153.	186	15.50	12	V
69	Schenkein HA, Loos BG. Inflammatory mechanisms linking periodontal diseases to cardiovascular diseases. *J Periodontol*. 2013;84:S51-S69.	183	30.50	17	I
70	Mustapha IZ, Debrey S, Oladubu M, Ugarte R. Markers of systemic bacterial exposure in periodontal disease and cardiovascular disease risk: a systematic review and meta-analysis. *J Periodontol*. 2007;78:2289-2302.	182	15.17	11	V
71	Paju S, Scannapieco F. Oral biofilms, periodontitis, and pulmonary infections. *Oral Dis*. 2007;13:508-512.	180	15.00	12	V
72	Iacopino AM. Periodontitis and diabetes interrelationships: role of inflammation. *Ann Periodontol*. 2001;6:125-137.	179	9.94	11	II
73	Ortiz P, Bissada NF, Palomo L, Han YW, Al-Zahrani MS, Panneerselvam A, Askari A. Periodontal therapy reduces the severity of active rheumatoid arthritis in patients treated with or without tumor necrosis factor inhibitors. *J Periodontol*. 2009;80:535-540.	179	17.90	27	V
74	Bartold PM, Marshall R, Haynes D. Periodontitis and rheumatoid arthritis: a review. *J Periodontol*. 2005;76:2066-2074.	177	12.64	8	I
75	Chávarry NGM, Vettore MV, Sansone C, Sheiham A. The relationship between diabetes mellitus and destructive periodontal disease: a meta-analysis. *Oral Health* Prev Dent. 2009;7.	176	17.60	19	III
76	Salvi GE, Yalda B, Collins JG, Jones BH, Smith FW, Arnold RR, Offenbacher S. Inflammatory mediator response as a potential risk marker for periodontal diseases in insulin-dependent diabetes mellitus patients. *J Periodontol*. 1997;68:127-135.	175	7.95	5	III

77	Dietrich T, Jimenez M, Kaye EAK, Vokonas PS, Garcia RI. Age-Dependent Associations Between Chronic Periodontitis/Edentulism and Risk of Coronary Heart Disease. *Circulation*. 2008;117:1668-1674.	174	17.40	12	V
78	Kamer AR, Craig RG, Dasanayake AP, Brys M, Glodzik-Sobanska L, de Leon MJ. Inflammation and Alzheimer's disease: possible role of periodontal diseases. *Alzheimers Dement*. 2008;4:242-250.	174	15.82	17	III
79	Iwamoto Y, Nishimura F, Nakagawa M, Sugimoto H, Shikata K, Makino H, Fukuda T, Tsuji T, Iwamoto M, Murayama Y. The effect of antimicrobial periodontal treatment on circulating tumor necrosis factor-alpha and glycated hemoglobin level in patients with type 2 diabetes. *J Periodontol*. 2001;72:774-778.	173	9.61	3	V
80	Schmidt AM, Weidman E, Lalla E, Du Yan S, Hori O, Cao R, Brett JG, Lamster IB. Advanced glycation endproducts (AGEs) induce oxidant stress in the gingiva: a potential mechanism underlying accelerated periodontal disease associated with diabetes. *J Periodont Res*. 1996;31:508-515.	172	7.48	7	V
81	Liu R, Bal HS, Desta T, Krothapalli N, Alyassi M, Luan Q, Graves DT. Diabetes enhances periodontal bone loss through enhanced resorption and diminished bone formation. *J Dent Res*. 2006;85:510-514.	171	13.15	11	IV
82	Stewart JE, Wager KA, Friedlander AH, Zadeh HH. The effect of periodontal treatment on glycemic control in patients with type 2 diabetes mellitus. *J Clin Periodontol*. 2001;28:306-310.	170	9.44	5	II
83	Pussinen PJ, Tuomisto K, Jousilahti P, Havulinna AS, Sundvall J, Salomaa V. Endotoxemia, immune response to periodontal pathogens, and systemic inflammation associate with incident cardiovascular disease events. *Arterioscler Thromb Vasc Biol*. 2007;27:1433-1439.	170	14.17	16	V
84	Taylor JJ, Preshaw PM, Lalla E. A review of the evidence for pathogenic mechanisms that may link periodontitis and diabetes. *J Clin Periodontol*. 2013;40:S113-S134.	170	28.33	19	III
85	Michaud DS, Liu Y, Meyer M, Giovannucci E, Joshipura K. Periodontal disease, tooth loss, and cancer risk in male health professionals: a prospective cohort study. *Lancet Oncol*. 2008;9:550-558.	169	15.36	23	III

86	Buhlin K, Gustafsson A, Pockley AG, Frostegård J, Klinge B. Risk factors for cardiovascular disease in patients with periodontitis. *Eur Heart J*. 2003;24:2099-2107.	168	10.50	1	III
87	D'aiuto F, Nibali L, Parkar M, Patel K, Suvan J, Donos N. Oxidative stress, systemic inflammation, and severe periodontitis. *J Dent Res*. 2010;89:1241-1246.	168	18.67	14	IV
88	Mikuls TR, Payne JB, Yu F, Thiele GM, Reynolds RJ, Cannon GW, Markt J, McGowan D, Kerr GS, Redman RS. Periodontitis and Porphyromonas gingivalis in patients with rheumatoid arthritis. *Arthritis Rheumatol*. 2014;66:1090-1100.	167	33.40	34	V
89	Seinost G, Wimmer G, Skerget M, Thaller E, Brodmann M, Gasser R, Bratschko RO, Pilger E. Periodontal treatment improves endothelial dysfunction in patients with severe periodontitis. *Am Heart J*. 2005;149:1050-1054.	165	11.78	6	III
90	Laine MA. Effect of pregnancy on periodontal and dental health. *Acta Odontol Scand*. 2002;60:257-264.	165	9.71	7	II
91	Mikuls TR, Payne JB, Reinhardt RA, Thiele GM, Maziarz E, Cannella AC, Holers VM, Kuhn KA, O'Dell JR. Antibody responses to Porphyromonas gingivalis (P. gingivalis) in subjects with rheumatoid arthritis and periodontitis. *Int Immunopharmacol*. 2009;9:38-42.	164	16.40	18	II
92	Guerrero A, Griffiths GS, Nibali L, Suvan J, Moles DR, Laurell L, Tonetti MS. Adjunctive benefits of systemic amoxicillin and metronidazole in non-surgical treatment of generalized aggressive periodontitis : a randomized placebo-controlled clinical trial. *J Clin Periodontol*. 2005;32:1096-1107.	164	11.71	11	III
93	Genco R, Offenbacher S, Beck JD. Periodontal disease and cardiovascular disease: epidemiology and possible mechanisms. *J Am Dent Assoc*. 2002;133:14S-22S.	163	9.59	2	II
94	Spahr A, Klein E, Khuseyinova N, Boeckh C, Muche R, Kunze M, Rothenbacher D, Pezeshki G, Hoffmeister A, Koenig W. Periodontal infections and coronary heart disease: role of periodontal bacteria and importance of total pathogen burden in the Coronary Event and Periodontal Disease (CORODONT) study. *Arch Intern Med*. 2006;166:554-559.	163	12.54	1	II

95	Demmer RT, Jacobs DR, Desvarieux M. Periodontal disease and incident type 2 diabetes: results from the First National Health and Nutrition Examination Survey and its epidemiologic follow-up study. *Diabetes Care*. 2008;31:1373-1379.	163	14.82	11	II
96	Lopez NJ, Da Silva I, Ipinza J, Gutiérrez J. Periodontal therapy reduces the rate of preterm low birth weight in women with pregnancy-associated gingivitis. *J Periodontol*. 2005;76:2144-2153.	163	11.64	4	V
97	Herrera D, Alonso B, León R, Roldán S, Sanz M. Antimicrobial therapy in periodontitis: the use of systemic antimicrobials against the subgingival biofilm. *J Clin Periodontol*. 2008;35:45-66.	163	14.82	16	V
98	Soskolne WA, Klinger A. The relationship between periodontal diseases and diabetes: an overview. *Ann Periodontol*. 2001;6:91-98.	157	8.72	8	IV
99	Dasanayake AP. Poor periodontal health of the pregnant woman as a risk factor for low birth weight. *Ann Periodontol*. 1998;3:206-212.	157	7.48	2	III
100	Rodrigues DC, Taba Jr M, Novaes Jr AB, Souza SL, Grisi MF. Effect of non-surgical periodontal therapy on glycemic control in patients with type 2 diabetes mellitus. *J Periodontol*. 2003;74:1361-1367.	156	9.75	2	V
